# Biopiezoelectric-based nanomaterials; a promising strategy in cancer therapy

**DOI:** 10.1186/s13046-025-03427-2

**Published:** 2025-06-04

**Authors:** Tingting Zuo, Saikat Dewanjee, Chao Zhang, Pratik Chakraborty, Wanxia Lu, Niraj Kumar Jha, Hiranmoy Bhattacharya, Moumita Gangopadhyay, Joshua Fleishman, Saurabh Kumar Jha, Zhe-Sheng Chen

**Affiliations:** 1 College of Biological Sciences and Technology, Yili Normal University, Yining, 835000 China; 2 Advanced Pharmacognosy Research Laboratory, Department of Pharmaceutical Technology, Jadavpur University, Kolkata, 700 032 West Bengal India; 3 Department of Neurosurgery, Zhujiang Hospital, Southern Medical University, Guangzhou, 510282 China; 4 Department of Biotechnology & Bioengineering, School of Biosciences & Technology, Galgotias University, Greater Noida, 203201 Uttar Pradesh India; 5 Department of Biotechnology, School of Life Science and Biotechnology, Adamas University, Barasat, 700126 Kolkata India; 6 Department of Pharmaceutical Science, College of Pharmacy and Health Sciences, St. John’s University, Queens, NY 11439 USA; 7 Department of Zoology, Kalindi College, University of Delhi, Delhi, 110008 India; 8 Centre for Himalayan Studies, University of Delhi, Delhi, 110007 India

**Keywords:** Cancer therapy, Electrical stimulation, Nanomedicine, Piezocatalysis, Piezoelectric materials

## Abstract

Cancer is one of the deadliest diseases, continually prompting physicians and researchers to investigate safe and effective modalities for its treatment. Piezoelectric nanomaterial is a new class of material with enormous potential for the nanoscale and bidirectional conversion of mechanical strain into electric fields for cancer treatment. In response to ultrasound mechanical strain, a piezopotential and electric field is generated in the tumor microenvironment, which reduces the growth of cancer cells by catalyzing redox reactions and the synthesis of reactive oxygen species. In this review, we discuss the basic concepts and mechanisms of biopiezoelectric nanomaterials as anti-cancer agents. We provide a comprehensive summary of current state-of-the-art piezoelectric nanoparticles as anti-cancer therapies. Lastly, we identify current challenges that must be addressed for the proper clinical development of biopiezoelectric nanomaterial-based anti-cancer agents and provide future perspectives for the development of this technology.

## Introduction

Malignant tumors are one of the most prevalent reasons for mortality worldwide [1, 2]. Current anti-tumor therapy is unsatisfactory, indicated by the high death rate and considerable metastases of malignant tumors [3]. Nanomedicine holds enormous promise for enhancing therapeutic efficacy while minimizing side effects [4, 5]. Recently, scientists have used the rapid evolution of nanomaterials (NMs) as inspiration for anti-tumor therapeutics. Piezoelectric (PZE) nanobiomaterials, which include ferroelectric, PZE, and pyroelectric materials, have recently gained a lot of attentions for anti-cancer applications [6]. In these materials, a charge dipole is induced in response to material-specific stimuli, e.g., mechanical strain, photon absorption, magnetic flux exposure, or heating, to produce a piezopotential and establish an electric field (EF) [7, 8]. Based on PZE theory, polarized charges can directly impact the tumor microenvironment, endorsing the formation of reactive oxygen species (ROS) [9, 10].

**Fig. 1:**
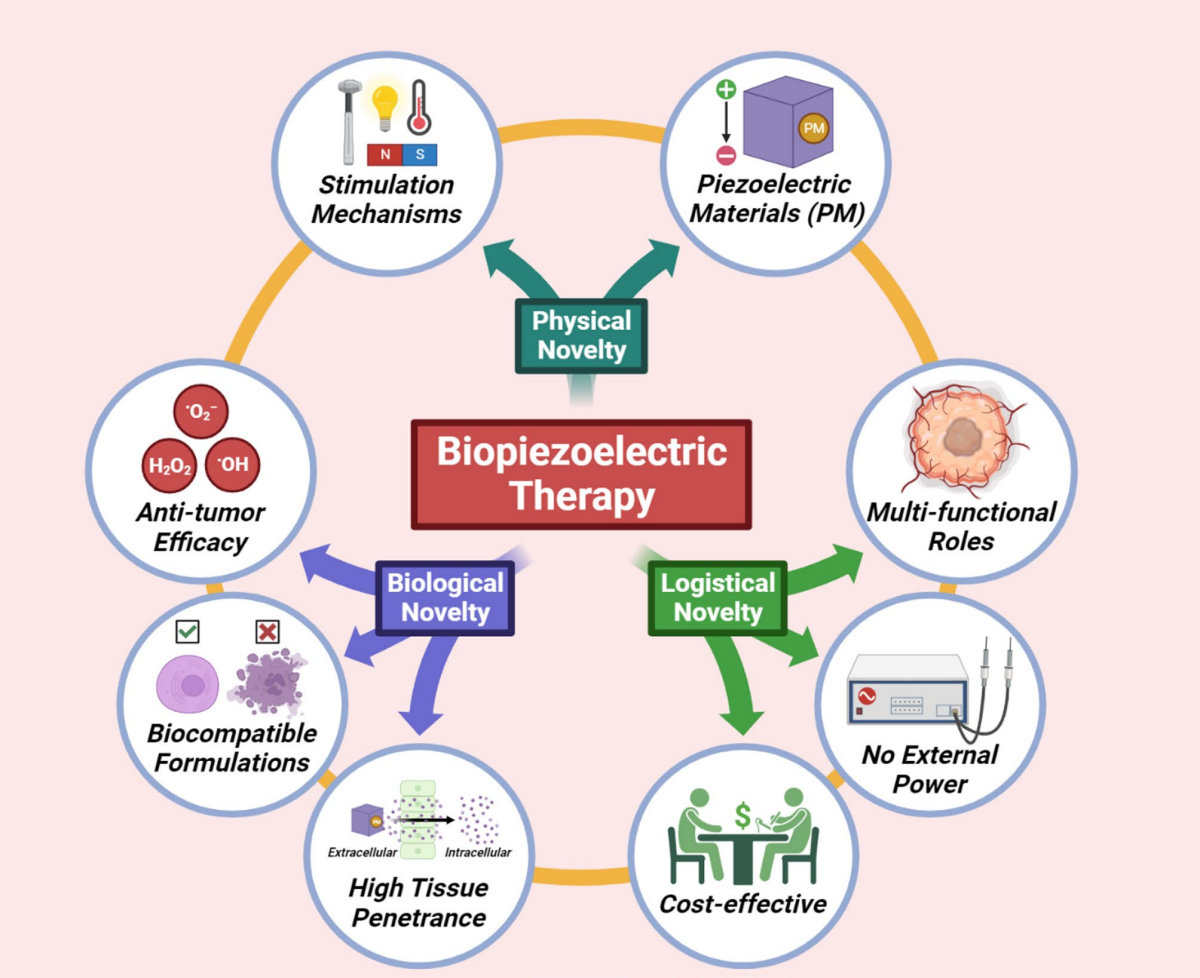
Key advantages associated with the physical, biological, and logistical novelties of biopiezoelectric therapy

The PZE effect is designated by the linear transition of mechanical and electrical variables. Electric dipole moments are created in PZE crystals by the asymmetrical arrangement of ions with different charges, creating two surfaces with opposing charge densities. Upon application of external strain to piezoelectric materials (PMs), the stretch between the positive and negative charge center is altered, leading to polarization aligned with the direction of applied strain. The direct piezoelectric effect (PZE_e)_ is created in this scenario when the surface-free charges are partly released to produce piezoelectricity [11]. PZE_e_ also includes the inverse PZE_e_, which is the conversion of an applied EF to crystal strain. Upon application of an EF in the direction of polarization of a PM, the material is mechanically deformed; however, deformation disappears when EF is withdrawn. This mechanism can be employed for signal excitation [12, 13]. PZE_e_ is incredibly relevant to biological systems, being exhibited in several biological tissues such as collagen and hydroxyapatite [14]. Of late, multidisciplinary research on bio-PMs (b-PMs) has garnered enormous interdisciplinary interests due to their phenotypical impact on biological systems and predicted biomedical applications (Fig. 1) [15, 16]. PMs offer anti-tumor promise along with exclusive tissue penetrability and biocompatibility in a cost-effective manner.

PZE therapy contributes significantly to the realm of nanomedicine by allowing for targeted, localized, wireless, and minimally invasive stimulation of cells and tissues, largely via ultrasound (ULS)-mediated PZE_e_ [17]. This method comes up with promise for a variety of applications, including targeted delivery, and tumor therapeutics. PZE therapy mostly utilizes ULS to activate nanoparticles and generate EF, offering promise towards localized stimulation and targeted therapy. The efficacy and possibilities of clinical translation rely on different aspects such as ultrasonic delivery optimization, and biocompatibility [18]. Photodynamic therapy uses light to activate photosensitizers, but is limited by its inability to treat tumor hypoxia. On the other hand, magnetic-based techniques, another non-invasive means of cancer therapy, can be useful for site-specific targeting, but may pose issues regarding tissue penetration and optimum magnetic strength. Interestingly, PZE therapy stands out to provide targeted electrical stimulation, potentially augmenting drug release. Further, it is also involved in the generation of ROS to destroy tumor cells.

In this review, we discuss biopiezoelectric nanoplatforms from the perspective of tumor management, summarizing recent developments an multiple potential strategies for improvement. In addition, we describe the mechanisms underpinning cutting-edge PZE biological platforms and provide much-needed insights for further anti-cancer therapeutic discovery.

## Prospects of piezopotential in tumor management

**Fig. 2:**
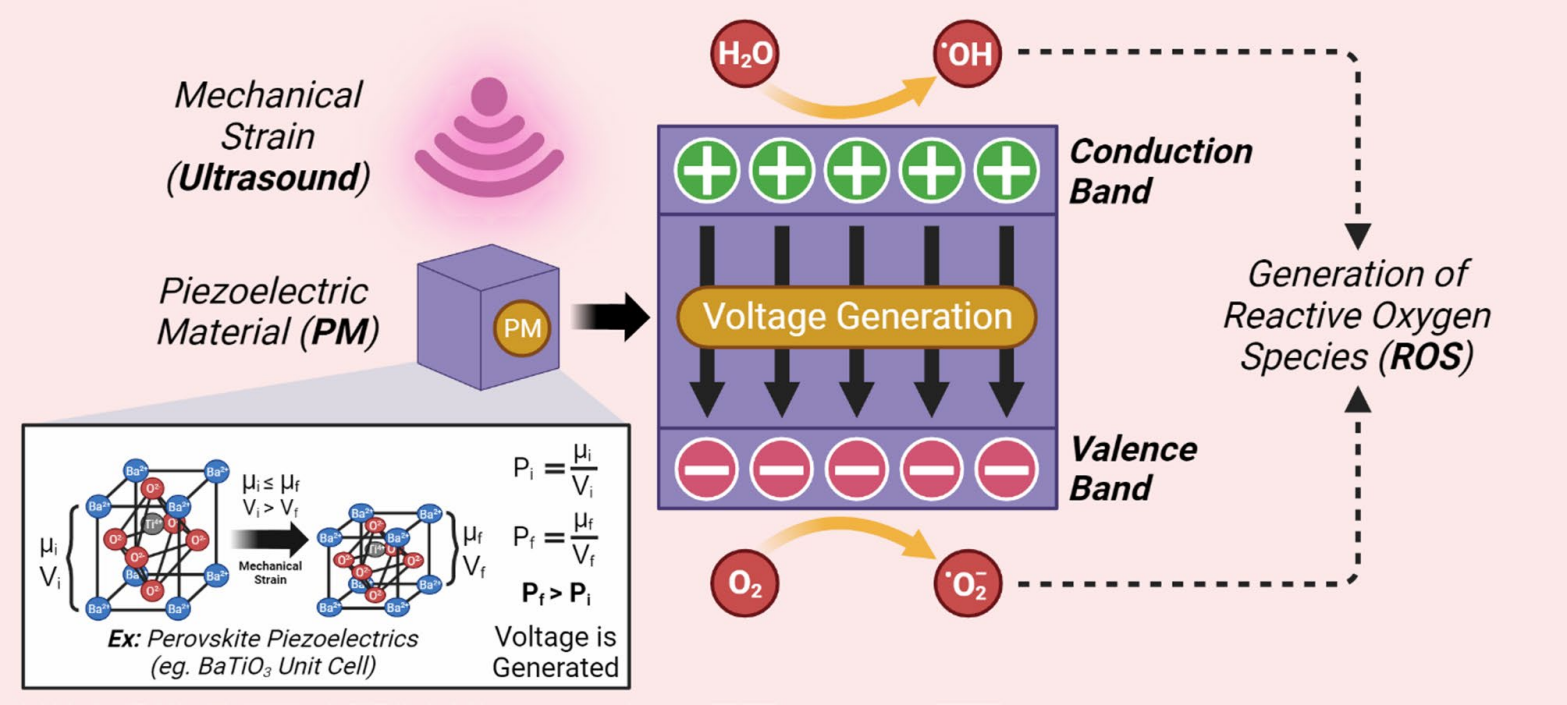
ULS-mediated piezocatalysis for the creation of NM dipoles, allowing for the catalysis of ROS synthesis. µ represents electrical dipole strength, V represents unit cell volume, and P represents polarization. Subscripts of i and f represent initial and final states respectfully. As a result, opposite surfaces exhibit heightened charge densities. The internal resultant EF changes electron band gaps allowing for the conducive formation of ROS

Any form of mechanical strain, such as those induced by ULS may deform a PM. Deformation removes the previously existing symmetry of NMs and polarizes positive and negative charges, creating a piezopotential in the direction of strain. Thus, charge density along surface of PM is proportional to the extent of the applied strain, which, when strong enough, piezo-electrocatalytically enhances ROS generation for anti-tumor treatment (Fig. 2). On removal of the mechanical strain, the material comes back to its ground state [19]. Holistically, this process allows for the conversion of mechanical strain into an EF, which is directed along the vector of strain. Thus, the direct PZE_e_ describes the process of transforming mechanical energy into electrical energy, a process termed mechano-electric transduction, which can be utilized for signal pick-up [20, 21]. Thus, PZE_e_ occurs through the interplay of dynamics and polarization.

### Promise of the PZE in cancer biology

Low-intensity electrical stimulation is emerging as a potential approach in cancer therapies. It has shown a great deal of promise in drug-independent inhibition of cancer cell proliferation [22, 23]. Low-intensity stimulation is thought to affect cell proliferation by interfering with the extracellular Ca^2+^ and K^+^ levels and degrading cellular elements necessary for cell division [24]. It also exhibits promise in reversing drug resistance associated with conventional chemotherapy. The b-PMs display excellent mechanical flexibility and low bio-toxicity. Organic PMs are often combined with inorganic PMs to form PZE composites [25]. As a consequence, both the high electromechanical coupling performance of inorganic PMs and the flexibility of organic PMs are restored, leading to an improved overall PZE performance. In addition, small dimensions enable them to speed up the rates of electron transfer and enhance the capacity of interacting with substrates. Hence, the dimension of PMs is critical for their biological performance [26]. PZE NMs (PNMs) appear to be viable platforms for the non-invasive and remote generation of local EFs within tissues. However, the lack of cellular specificity and documented changes to the replication of non-malignant surrounding cells are emerging challenges that require serious consideration [27].

The standard therapeutic strategy for tumor management involves surgical excision followed by radiation and chemotherapy. However, the appearance of adverse effects, and the advent of drug resistance are frequently encountered with the conventional clinical protocol of tumor treatment [28]. Over the last few years, progress has been made toward anti-cancer therapies, notably those in the drug nanodelivery systems [5, 29]. However, conventional nanoformulations suffer from multiple limitations (Table 1), inspiring researchers to develop novel and roundabout strategies to overcome them [30]. Some advanced nanosystems have been designed over the last few years. Among them, PNMs come up with exciting promises, particularly in the field of cancer management.

**Table 1:** Limitations of conventional anti-cancer nanoparticles (NPs)

Delivery systems	Constraints
Polymeric NPs	Limited loading and encapsulation efficiency, chances of aggregation, and residual materials
Metal-based NPs	Thermodynamic instability, risk of bioaccumulation
Liposomes	High cost, short lifespan, storage constraints to protect phospholipids from oxidation and hydrolysis
Solid lipid NPs	Low drug loading capacity, chances of aggregation
Niosomes	High cost, prone to oxidative degradation

PNMs interact with biological systems mainly through two mechanisms. One involves direct electrical stimulation of the biological system, impacting growth, development, proliferation, and other cellular behaviors regulated by membrane potentials e.g., ion-gradients and electro-sensitive proteins. The other is related to piezo-induced ROS generation, endorsing oxidative stress, the activation of the apoptotic cascade, and allowing for potential use in tumor therapies [31, 32]. PMs can be functionalized into b-PMs, which can selectively interact and communicate with certain tissues through surface modification and material engineering techniques. Recent advancements have opened new avenues regarding b-PMs which combine outstanding biocompatibility with excellent PZE performance [33]. The energy transition from mechanical to electrical energy within biological systems has been successfully achieved with the discovery of biocompatible PMs like barium titanate (BaTiO_3_), polyvinylidene fluoride (PVDF), and potassium sodium niobate (KNN), leading to the development of novel b-PMs for diverse biomedical applications, including tumor therapy [34, 35]. Further, PNMs can be stimulated wirelessly by external energy sources like ULS, which potentially allow scopes to remove the limitations associated with mainstream electrical stimulation techniques [36, 37].

PZE-induced electrical stimulation has the potential to overcome issues regarding both resistance and recurrence [38]. Earlier studies have shown that electrical stimulation decreases drug resistance by interfering with the P-glycoprotein efflux process, which is involved in the pumping of chemotherapeutic drugs out of tumor cells [39, 40]. Owing to ease of operation and/or application, good tissue penetration depth, and minimum tissue injury, ULS is the most extensively employed mechanical strain in cancer theranostics [41]. Under wireless ULS strain, TiO_2_-BaTiO_3_ nanorods can promote intracellular ROS generation by piezocatalysis, promoting cell death [42] Mechanistically, ULS provides compressive/tensile strain on PMs, allowing for the generation of EFs that separate free electrons and holes. Improved redox activity due to free electrons/holes interacting with water and molecular oxygen to produce ROS [43, 44]. As a result, PZE sonosensitizers often display high efficiency regarding ROS generation. This process, resembling PZE catalysis is also termed as PZE-catalyzed dynamic therapy [45].

Pyroelectric dynamic therapy employs pyroelectric materials. When heated, pyroelectric materials generate a charge dipole and, from such, induce ROS generation for anti-cancer therapy [46]. Experiments have revealed that a strong EF enhances membrane permeability [47, 48]. The development of controlled drug delivery systems is a proven strategy for increasing drug bioavailability and reducing side effects [5, 49, 50]. The intrinsic polarization ability of PZE carriers in response to mechanical strain has demonstrated strong benefits on drug binding and release characteristics [51].

PMs demonstrate promise regarding the creation of multi-functionalized drug carriers to address the challenges regarding systemic toxicity, targeted administration, enhanced internal transport stability, and postoperative relapse associated with standard chemotherapeutic protocols [52]. It is essential to note that surface charge has a significant influence on lowering cell membrane integrity and inducing cell death [53, 54]. Among the significant inventions in this field, a PZE single-crystal acoustic transducer has been designed to endorse endoscope-mediated drug release at the desired part of the digestive tract, providing an alternative option for unresectable tumors [55]. Mushtaq and colleagues [56] developed PZE nanorobots with the ambition of effective delivery of chemotherapeutic agents to cancer cells. Nano-PZE immunotherapy activates the immune system by modulating electrical impulses generated by PNMs, emerging as a promising tool in anti-tumor therapy [57]. Electrical signals bear the potential to regulate the activities of immune cells directly, opening up an array of biomedical applications [58]. Discovering quick and effective ways to induce phenotypic polarization toward proinflammatory macrophages may aid in the elimination of cancer cells or restrain the development of tumors through an inflammation-mediated pathway. Relying on micro-vibrational deformations of PZE films, Kong and colleagues [59] used local electrical signals in conjunction with ULS to non-invasively enhance the proinflammatory response of macrophages.

### Biocompatibility

Two major factors define the effective application of a PM in biology: biocompatibility and biosafety. For biopiezoelectric nanomaterials (b-PNMs), the biodegradability and excretion rates are generally verified to avoid bioaccumulation in the body [60], the degradation of such being heavily influenced by the physiological environment [8, 61]. Most b-PMs are effectively cleared from the system through a degradation process, and in some cases, the ions arising as degradation products also impart therapeutic benefits [8, 15]. Many developed b-PMs have exhibited acceptable biocompatibility and biodegradability. Interestingly, ferroelectric materials display higher biocompatibility than ferromagnetic components, further taking the debate in favor of b-PMs [62]. Surface modification emerged as a potential approach to improve the biocompatibility as well as the pharmacokinetic profile of PMs [63]. Inclusion of polymeric coatings, such as poly(D, L-lactide-co-glycolide), poly(glycolic acid), and poly(lactic acid) have improved the biocompatibility and biodegradability of PZE agents [64]. Non-biodegradable materials may be PEGylated and/or be made electrically neutral by controlling surface characters, achieving a stealth effect [65].

Factors like morphology, surface charge, aspect ratio, and surface functional groups influence tumor penetration and clearance time [66]. Metabolism and excretion by the liver and kidney are both affected by the physicochemical properties of b-PMs, e.g. size, morphology, surface charge, etc [67]. The rate of elimination largely depends upon surface charge whereas the extent of renal excretion is determined largely by particle size [68]. The mechanism of clearance relies upon the interaction of inorganic materials with intracellular components. Materials of > 200 nm are filtered in the spleen and subsequently cleared via the reticuloendothelial system [69]. The prerequisite for PMs to be utilized as nanomedicines is to meet the size-range of biological metabolism. Health authorities are taking necessary measures to evaluate the potential toxicological aspects to assure the biosafety of PZEs [70]. The process further calls for close collaboration among different stakeholders i.e. investigators, clinicians, and regulators unanimously.

## PZE biomaterials at a glance

An ideal PM is biocompatible and easily coupled for electromechanical activity, providing optimal activity within biological systems [25]. There may also be a requirement for stimuli-responsiveness, the ability to respond to physiological activity, strength, and mechanical flexibility. Current b-PMs can broadly be categorized based on their chemical structures. Major characteristics of representative materials from each class are enlisted in Table 2.

**Table 2:** Frontrunners among b-PMs for anti-tumor therapeutics

Materials	Class	Structural features	Properties
BaTiO_3_	PZE ceramic	Perovskite structure	High electromechanical coupling ability, low dielectric loss, good biocompatibility
KNN			Superior PZE co-efficient, good biocompatibility
ZnO		Wurtzite structure	High electron mobility, good biocompatibility
PVDF and its copolymers	PZE polymer	β, γ, and δ crystal structures are electroactive	Good flexibility, ease of processing, good biocompatibility
Glycine	PZE biomaterial	Achiral	High biocompatibility
Diphenylalanine		Non-centrosymmetric hexagonal crystal	Stable PZE property, high biocompatibility
Collagen		Triple helix	High biocompatibility (abundantly found in mammals)
MoS_2_	Atomic scale PZE material	Only the odd-layered crystal is PZE, owing to broken inversion symmetry	Strong redox capacity

The PZE characters of ceramics are intimately related to their crystal structures. Pervoskite-type b-PMs are widely used in biomedical fields owing to high PZE activity, good electromechanical coupling, and low cost of manufacturing [71]. Lead-free, perovskite-structured materials e.g. BaTiO_3,_ BiFeO_3,_ LiNbO_3_, niobates, and titanates offer better biocompatibility with good electromechanical coupling [72]. Interestingly, many non-ferroelectric materials, e.g. ZnO, AlN, CdS, CdSe, GaN, and InN, exhibit biopiezoelectric properties owing to the presence of wurtzite crystal structures [73]. Owing to their biocompatibility, fine nanostructures, and high voltage electroactivity, these semiconductors bear the potential to be used in biomedical fields [15, 74]. Despite their exceptional PZE capabilities, PZE ceramics contain few intrinsic drawbacks, such as stiffness, brittleness, and requirement for high-temperature processing. As a result, biopiezoelectric ceramics offer low mechanical tolerance towards defects and external strains. Majority of the biopiezoelectric sensors and transducers, like wearable biosensors, implanted devices, place a significant demand regarding material flexibility [75]. However, ceramic b-PMs with low fracture toughness are prone to fracture upon exposure to mechanical loads or flexure, limiting the potential for biomedical applications. In order to overcome such limitations, biopiezoelectric ceramics may be put together into ultra-thin films to improve mechanical flexibility [19, 76].

Certain flexible polymers display piezoelectricity and ferroelectricity upon stretching. Owing to excellent mechanical flexibility, lightweight, low dielectric constant, simplicity of manufacturing using low temperature, and ease of large-scale integration, piezoelectric polymers (PPs) are counted among highly appealing b-PMs for biomedical purposes [77]. Optimization of certain process parameters allows for precise customization of their optical transparency and electromechanical coupling efficiency [8]. Most importantly, bio-PPs possess low toxicity and acceptable biocompatibility, including some biodegradable examples such as poly-3-hydroxybutyrate-3-hydroxy valerate [78]. Bio-PPs are intriguingly becoming one of the foremost choices for the development of miniature, flexible, bioimplants and sensing devices due to their structural flexibility, manufacturing simplicity, high chemical stability, outstanding biocompatibility, and mechanical strength. PVDF and its copolymers offer good prospects towards self-powered flexible PZE sensors and/or energy harvesters, with a wide range of potential biomedical applications. However, compared to inorganic PZEs, most bio-PPs typically exhibit relatively low PZE charge coefficients, resulting in lower levels of charge generation [78, 79]. Emerging evidence proposes that the PZE constant of PVDF-based flexible PZE sensors can be improved by increasing the β-phase content, crystallinity, and molecular chain orientation of PVDF and associated copolymer films, or by manufacturing them with inorganic PMs [8, 80, 81]. Biopiezoelectric composites can overcome the temperature constraint of bio-PPs as well as the intrinsic brittleness of inorganic biopiezoelectrics [82]. For instance, biopiezoelectric ceramics and polymers have been effectively coupled to produce devices with synergistically improved mechanical flexibility, biocompatibility, and piezoelectricity, thereby providing an attractive biopiezoelectric composite for various biomedical usage including anti-cancer potential [83].

Certain biomaterials can give rise to PZE_e_ due to their minimal symmetry and lack of an inversion center [84]. These organic b-PMs are intriguing to cancer researchers owing to high biocompatibility, constant PZE coefficients, and acceptable dielectric characters [6, 85]. Amino acids represent non-centrosymmetric crystal structures, and exhibit ferroelectric characters. While peptides and proteins are composed of amino acids as the fundamental units, amino acid sequences and their spatial conformations provide structure-dependent PZE properties to them. For example, the PZE charge coefficient of β-glycine, containing a non-centrosymmetric crystalline structure is comparable to that of inorganic PMs [78]. In most cases, upon exposure to mechanical strain, dipole moments of amino acid residues reorient longitudinally, resulting in an alteration of polarization, and PZE response [78, 86]. Interestingly, several phytoconstituents including lignocellulosic compounds display PZE responses [87]. In this context, nanoscale cellulose molecules have the potential as futuristic b-PMs for biosensors, actuators, and other biocompatible devices in cancer management.

Interestingly, common non-PMs often exhibit piezoelectricity when they reach specific nano or sub-nano dimensions [6, 88]. One of the most crucial structural characteristics of NMs is the surface effect brought about by virtue of large specific surface area [89]. The ratio of surface atoms to all other atoms increases significantly as the size of the material goes down. The existence of dangling bonds and defects on the surface dramatically changes their electrical, optical, magnetic and catalytic features from those of macroscopic bulk materials [53].

## Novel biopiezoelectric nanoplatforms for cancer therapy

The b-PNMs attract a lot of attention for anti-tumor applications. Polarized charges are formed in the PNMs, resulting in piezopotential and a built-in EF. The polarized charges can directly influence materials in the tumor microenvironment, assisting in carrier separation and inhibiting recombination [8]. Upon application of external strain, PMs generate PZE potential which, in turn, interferes with ion channels and subsequently impedes proliferation of cancerous cells. The anti-cancer efficacy of b-PMs is primarily regulated via PZE stimulation, ROS production, and accuracy and precision regarding drug delivery. Progress in nanoscience has opened up new possibilities regarding the production of novel b-PNMs. Compared to their bulk counterparts, b-PNMs offer several advantages for biomedical applications. They can effectively pass through several physiological barriers, including blood vessels and cell membranes. Moreover, PZE nanocatalysts frequently display higher catalytic effectiveness than bulk catalysts for piezocatalysis-based applications, which increase their electron transfer rate and strengthen the contacts with substrates [90].

All 0D, 1D, and 2D b-PNMs with both PZE properties and nanosize effects exhibit significant potential for a wide range of biomedical applications including cancer management. 0D b-PNMs, such as BaTiO_3_ NPs (B-NPs) demonstrate good biocompatibility and quick metabolic rates [91]. Furthermore, the formation of nanoclusters by assembling 0D b-PNMs with polymer films or quantum dots (QDs) can improve PZE characteristics [92]. 1D b-PNMs display better charge transfer efficiency compared to 0D b-PNMs [93]. Furthermore, they can overcome the agglomeration issues that plague many 0D b-PNMs [94]. Also, 1D b-PNMs display excellent processability, outstanding PZE properties, good sensitivity, and high flexibility [15]. The majority of 1D b-PNMs are biocompatible, which enhances their potential for widespread use [95]. Interestingly, 2D nanosheets have drawn a lot of attention in the field of biomedicine over 1D b-PNMs due to their ability to eliminate the incidence of fractures on exceeding the critical force limit, a problem frequently associated with 1D materials. Further, they can alter the redox homeostasis in cancer cells, which enhances their appeal in cancer therapeutics [41]. When thinned to a nanoscale thickness to generate a 2D shape, PMs (as well as conventional PNMs) can lose centrosymmetry in one direction and display increased piezoelectricity [53, 96]. The planar 2D b-PNMs exhibit a flexible shape and induce surface polarization with the application of external strain. Progressing with b-PNMs over the past few years, 3D printing technology has gained considerable attention in fabricating PMs with improved efficiency and structural diversity [97].

Given that mechanical characteristics and geometry are linked by a strong correlation, a well-designed and optimized morphology can impart a favorable impact on the overall PZE response [15, 98]. Nanowires with elongated polar axes have exhibited better pyroelectric performance compared to equiaxed NPs, wherein maximum output voltage was reported to be directly correlated with the length of nanowires [99, 100]. The polarization effect of pyroelectric catalysts increases with increase in particle size, and alterations in size-dependent phase composition and domain structure [101]. Apart from morphology and dimension, the initial volume contraction due to residual surface stress and the surface piezoelectricity also contribute to the improvement of the PZE coefficient [102].

PZE single crystals tend to deliver better performance compared to PZE ceramics, which comprises of the same components since they are not affected by grain boundaries, grain size, and void fraction [103]. Thus, single crystallization emerges as an efficient technique to improve PZE performance. On the contrary, single crystals are costly and difficult to produce on large scales. Consequently, textured synthesis of PMs has evolved as a probable alternative [104]. Doping and compositing are promising approaches to enhance the PZE performance in addition to morphological tweaking. Increases in the PZE coefficient and the dielectric constant are the main benefits of doping and compositing [105, 106]. Compositing with a polymer can also confer superior flexibility and stretchability, eventually improving output performance and durability [107].

Despite the immense promise of b-PNMs, preparing good-quality nanostructures with well-defined morphologies remains a considerable challenge. Mechanical exfoliation is a simple and fast physical separation approach for the synthesis of b-PNMs. However, it comes up with several intrinsic limitations, including product inhomogeneity, limited stripping effectiveness, and poor control over morphology of NMs [108]. Liquid exfoliation technique has emerged as an extension of mechanical exfoliation. By forming a protective layer on the surface of the lamellar products, the solvent molecules are enabled to inhibit re-stacking and aggregation [109]. Solvothermal and hydrothermal syntheses have proven to be effective approaches for producing b-PNMs with a wide range of morphologies [110]. These techniques, with their low cost, simple one-pot operation, low reaction temperature, and diverse applicability towards different sizes, morphologies, and dimensions may produce hierarchical nanoparticulate structures with specific geometry. Electrospinning is a typical method for preparing nanofibers [111]. By controlling the electrospinning variables and the composition of precursor solution, morphology resembling natural tissue can be induced within biopiezoelectric nanofibers [112]. Chemical vapor deposition is another common technique for producing b-PNMs with acceptable purity, good crystallinity, and tunable thickness. However, the formation of sulfur (S) vacancies during the process limits PZE capabilities. Passivation of S vacancies through S-treatment can effectively overcome this gap to improve PZE performance [113]. Altering the temperature and applying external EF can enhance the polarization intensity (that increases with temperature and EF), thereby boosting redox reactions [114].

Ideal b-PMs should demonstrate not only excellent PZE performance but also adequate surface qualities e.g. hydrophilicity, roughness, and porosity customized to their biological application. PNMs should be biocompatible and/or biodegradable to ensure biosafety and possess suitable mechanical characters to enable long-term durability. During redox reactions, the adsorption of reactants and the separation of charge carriers are critical to produce free radicals. Modification of surfaces and interfaces also improve material polarization. Thus far, main strategies for modulating the surface and interface of materials include surface modification, surface conditioning, precious metal deposition, and heterostructure building [8, 115]. Owing to the plasma effect and high functionalities of metals, the deposition of noble metals on PZE surfaces can improve charge separation efficiency, leading to remarkable redox reaction performance [116]. The selection of semiconductors with satisfactory energy band structures to construct heterojunctions seems to emerge as more suitable strategy compared to the utilization of noble metals to intercept the compounding of positive and negative charges produced at elevated temperatures, and to extend the charge lifespan [117]. For biomedical applications, the surface characteristics of b-PMs are critical factors. For instance, the porosity of scaffolds governs the cell penetrability, while surface roughness encourages cell adhesion [118]. The surface wettability can also control cell attachment and spread during tissue regeneration. In contrast, a high hydrophobicity impedes cell attachment and expansion, thus limiting therapeutic applications as observed with PZE PVDF polymer. However, the formation of a super-hydrophilic surface on PVDF scaffolds by plasma treatment can promote the adhesion and diffusion of cells [119].

## Applications of b-PNMs in cancer therapeutics

**Fig. 3:**
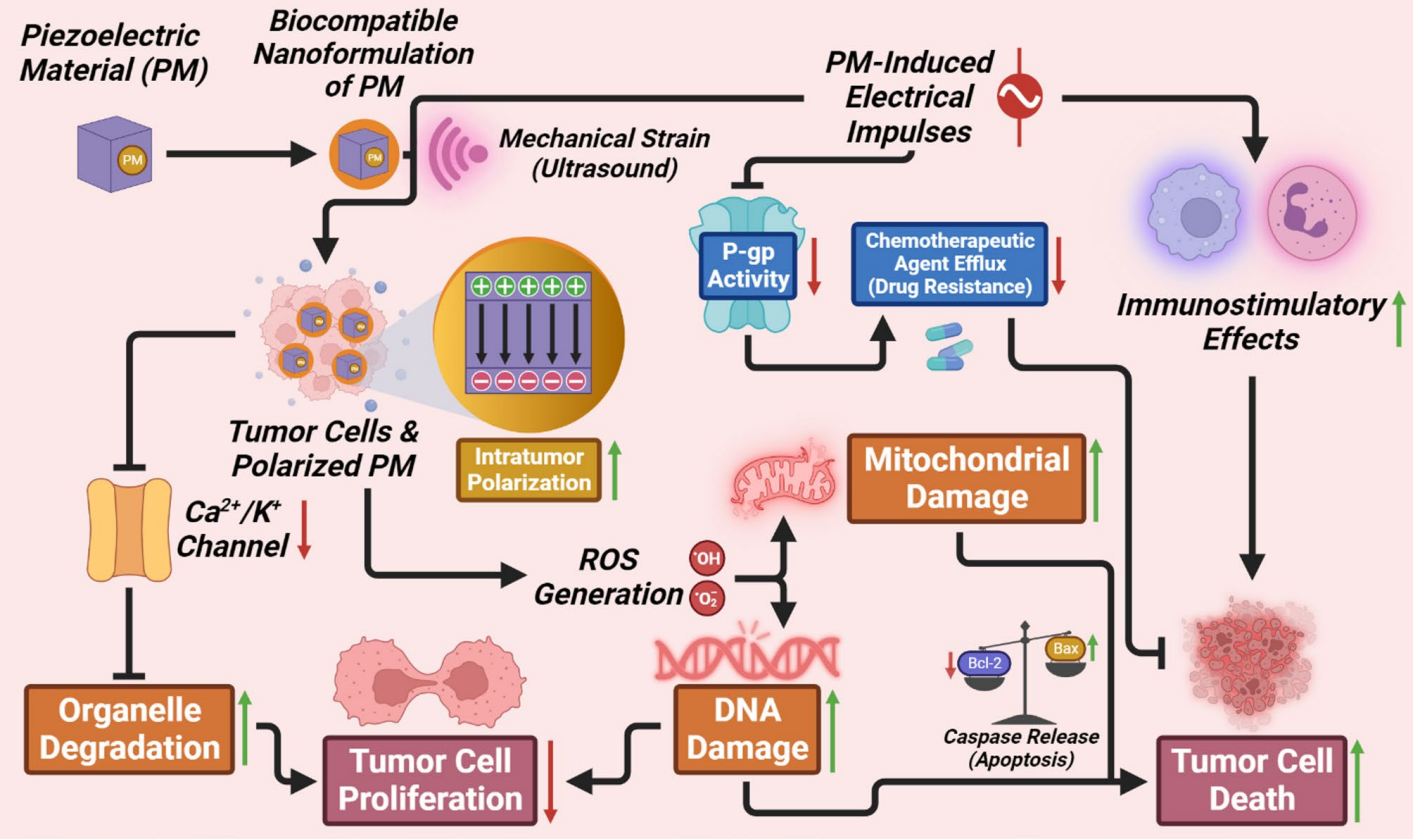
Schematic representation of biopiezoelectric-based tumor targeting, triggered by ultrasound mechanical strain

The anti-cancer efficacy of b-PMs is essentially mediated through PZE stimulation, ROS generation, and drug delivery. On application of external strain, PMs generate PZE potential which, in turn, interferes with ion channels and inhibits cancer cell proliferation [120]. Local and target-specific delivery of electric cues selectively to cancer cells is exceedingly imperative to avoid undesirable stimulation to healthy cells. Fig. 3 depicts the anti-cancer mechanisms induced by PZE substances in tumor cells. Table 3 enlists application-oriented outputs of different b-PNMs in tumor management.

**Table 3:** Applications of biopiezoelectric nanoplatforms in cancer treatment

PZE materials	Nano-platforms	Implications	Operational concepts	Types of cancer	Outcomes	References
Au-coated ZnO	Nanorods	Chemocatalytic and piezocatalytic activity	ULS-triggered, PZE-mediated ROS formation, delivery of piperlongumine	Breast cancer	Cancer-specific chemo-piezocatalytic combination therapy	[121]
Au NP-conjugated calcium phosphate	Nanowires	Piezocatalysis	ULS-mediated ROS generation	Mouth epidermal carcinoma	Enhanced piezocatalysis, intracellular ROS generation for wireless cell therapy to selective cancer cells	[122]
BaTiO_3_	NPs	PZE potential	Generation of ULS-activated electrical signal to cancer cells	Breast cancer	Antibody-based target specificity, piezopotential-based obstruction to cell proliferation	[24]
				Glioblastoma multiforme	Dual targeting-mediated increased sensitivity, anti-proliferative and pro-apoptotic effects	[22]
	Tetragonal NPs		Sonodynamic therapy	Triple negative breast cancer	Stable sensitization and dynamic control of redox reaction outcomes	[45]
	NPs		Internal wireless electrical stimulation		Regulation of cell proliferation and migration	[123]
	PZE nano-prodrug	Piezocatalysis, chemotherapy	ULS-triggered piezocatalysis, chemodrug release	Pancreatic ductal carcinoma	Efficient tumor targeting, controlled release of camptothecin	[124]
	NPs	Photothermal, photodynamic, catalytic therapy	Oxygen-independent synergistic activity	Triple negative breast cancer	High therapeutic efficacy even in hypoxic conditions	[125]
		PZE potential	Production of ROS and oxygen by ULS-triggered piezoelectricity		Downregulation of tumor growth and metastasis, and angiogenesis	[126]
		Immunotherapy	Activation of MMP2 for PDL-1 blockade, piezocatalysis, ROS generation, water splitting	-Melanoma	Enhanced immune response against tumors	[127]
		Breaking of intracellular bioelectric homeostasis	Inhibition of eNOS/NO pathway of angiogenesis	Melanoma	Normalization of tumor vasculature, improved therapeutic efficacy by combined chemotherapy and PZE therapy	[128]
Bi_2_Fe_4_O_9_	Nanosheets	Nanozyme therapy	Temperature-dependent enzymatic kinetics	-Triple negative breast cancer	Induction of apoptosis and ferroptosis, with minimized off-target toxicity	[129]
BiOCl	Nanocatalyst	Piezo and radio catalysis	Piezocatalysis under ULS excitation followed by radio-catalytic hydroxyl radical generation	Triple negative breast cancer	Piezo and radio catalytic tumor suppression	[130]
Black phosphorus	Nanosheets	Sonodynamic therapy	ULS irradiation with different output power densities and time	Triple negative breast cancer	PZE polarization-mediated ROS production devoid of off-target toxicity	[131]
CdS-assisted nano-Nb_2_C	Nano-lymphatic	Pyroelectric catalysis	Reduction in tumor interstitial pressure	-Cervical cancer	Improved tumor penetration, lower proliferation, and recurrence	[132]
CoFe_2_O_4_–BiFeO_3_	NPs	Magnetoelectrodynamic therapy	Initiation of intratumoral magneto-driven, PZE-catalyzed reaction by magnetostrictive-PZE NPs	Triple negative breast cancer	Excellent catalytic dynamic responsiveness, good therapeutic efficacy, and biosafety	[133]
Cu_2 − *x*_O-BaTiO_3_	PZE heterostructure	Piezocatalysis	Multifunctional dynamic therapy	Breast cancer	Integration of sonodynamic therapy and chemodynamic therapy to improve therapeutic efficacy	[134]
MnTiO_3_	Nanosheets	Piezodynamic therapy	ULS-activated enzymatic activity	Triple negative breast cancer	Programed catalysis of tumor death	[135]
MoS_2_	Nanoflowers	PZE potential	Generation of ULS-activated electrical signal to cancer cells	Skin cancer	> 80% tumor reduction in 7 days via caspase 3-mediated apoptosis	[136]
	Self-assembled multi-modal nanoplatform	Nanozyme therapy	Piezoelectricity-mediated ROS production under ULS influence	-Triple negative breast cancer	Optimum anti-tumor efficacy with negligible toxicity to normal tissue	[137]
PVDF	Nanoeels	Drug delivery	Magnetically controlled PZE nanorobot	Breast cancer	Magnetic manipulation of locomotion, and pulsatile release of doxorubicin	[56]
	Nanofilms	Mechanoelectrical therapy	Restriction to cell proliferation by mechano-electrical stimuli	Rhabdomyosarcoma	Promising improvements regarding skeletal muscle cancer	[21]
PVDF-TrFE	Lipid-polymer hybrid nanostructures	Drug delivery	Remote ULS stimulation induces nutlin-3a release, and aids in transmission of electrical signals locally	Glioblastoma multiforme	ApoE-mediated passage through BBB, activation of apoptotic and anti-apoptotic pathways in drug-resistant tumor cells	[138]
	ApoE-functionalized polymeric PZE NPs		Chemotherapy, PZE stimulation	Brain cancer	Nutlin-3a and ULS-mediated PZE potential restricts the angiogenic activity by microvascular endothelial cells of the brain	[139]
Selenium-doped KNN	Implant	Wireless electrochemotherapy	ROS formation, mitochondrial damage, induction of caspase 3 pathway	Osteosarcoma	Enhanced apoptosis, good biocompatibility	[140]
Sphalerite	Nanosheets	Piezophotocatalysis	Intracellular ROS burst leading to apoptosis	Breast cancer	Induction of apoptosis of cancer cells	[32]
WS_2_	Nanosheets	ULS-mediated piezodynamic tumor therapy and energy metabolism-targeted chemotherapy	PZE potential, disruption of energy metabolism	Breast cancer	Effective modality of tumor treatment devoid of systemic toxicity	[141]
β-PVDF	Nanofilms	Immunotherapy	Regulation of macrophage polarization with localized electrical signals	Leukemia	Electrogenetic regulation of immune cells to restrict tumor cell proliferation	[59]

### BaTiO_3_ biopiezoelectrics and successors

BaTiO_3_, due to its strong PZE capabilities and great biocompatibility, has gained a lot of attention in cancer therapy. Marino and colleagues [24] formulated an ULS-responsive novel PNM for cancer treatment; B-NPs generated a PZE potential in response to ULS strain, which substantially reduced the proliferation of breast cancer cells by interfering with K^+^/Ca^2+^ homeostasis. Additionally, these NPs were tagged with anti-HER2 antibodies to precisely target HER2-expressing breast cancer cells. Thus, it exemplifies the synergistic potential of both ULS-aided PZE-based cancer therapies and existing targeted approaches. In another relevant experiment, H_2_O_2_ was exploited to hydrophilize tetragonal B-NPs before embedding them in an injectable hydrogel, which endorses ROS formation under ULS stimulation [45]. To utilize the efficient generation of ROS, researchers designed tetragonal BaTiO_3_ PNMs, that, in turn, catalyze the formation of ROS to impart cytotoxic effects for tumor cell eradication via ULS-triggered catalytic effects. The built-in EF can change the band alignment to make the formation of toxic ROS energetically favorable, as per the finite element modeling simulation. Cumulatively, injectable NPs-embedded thermosensitive hydrogels offer astonishing biosafety profiles in addition to considerable cytotoxic effects on tumors.

The PZE-based strategy offers fascinating advantages over traditional sonoluminescence-activated sonodynamic therapy, imparting more stable sensitization of tumor cells and a higher degree of control over redox reaction outcomes. As an example, internal wireless PZE stimulation from B-NPs has shown a non-invasive treatment for triple negative breast cancer [123]. Interestingly, the study decoded a favorable correlation between the anti-cancer impact and the intensities of electric stimulation. Internal electric stimulation from PNMs downregulated the proliferation and migration of cancer cells. Recently, PZE sonosentitizers have been designed for multifunctional dynamic therapy for breast cancer [134]. Herein, Cu_2 − *x*_O-BaTiO_3_ PZE heterostructures were formulated to simultaneously act as sonosensitizer and chemodynamic agent, to improve ROS production, and subsequently improve anti-cancer outcomes. Under ULS influence, the heterojunction provides a high-performance production of singlet oxygen (^1^O_2_) and hydroxyl radicals (•OH). It also depicts Fenton-like reaction activity, converting endogenous H_2_O_2_ into hydroxyl radical for chemodynamic therapy. This research integrates sonodynamic and chemodynamic therapy by employing PZE heterostructures to serve as acoustic sensitizers as well as chemokinetic agents, considerably improving therapeutic efficiency against cancer, and serving as notable reference for the novel application of piezocatalysts.

In search of alternative piezocatalyst materials, Dolai and colleagues [122] discovered Au-NPs conjugated calcium phosphate nanowires as piezocatalysts. Their anisotropic shape and conjugation with Au-NPs augmented the piezocatalytic capability of NMs by up to 10 times. The method can be utilized to form a colloidal nano-bioconjugate for selective labeling of cancer cells, followed by wireless cell treatment using medical-grade ULS-based intracellular ROS production. PZE black phosphorus nanosheets, a novel group of sensitizers for sonodynamic therapy has exhibited high efficacy in halting tumor growth [131]. Ultrasonic waves induced mechanical strain in the 2D nanosheets, leading to PZE polarization. During ULS therapy, the intrinsic energy band of black phosphorus was tipped, and hence the negativity of the conduction band of black phosphorus was greater than that of oxygen radicals, accelerating ROS formation. The work under discussion identifies a new mechanism for sonodynamic sensitizers in addition to suggesting 2D black phosphorus as an excellent sensitizer towards the sonodynamic therapy of tumors. Ge et al. [133] combined nanocatalytic chemistry and magnetic physics to evaluate core-shell-structured CoFe_2_O_4_-BiFeO_3_ magnetoelectric NPs. Magnetostrictive cores and multiferroic PZE shells integrated with core-shell-structured magnetoelectric NPs, forming a magneto-electric engine to generate positive and negative polarization charges on the surface. It accelerated the oxidation of water and the reduction of oxygen, leading to the generation of ROS in the forms of hydroxyl and superoxide radicals, which in turn induced both in vitro cell death and in vivo ablation of local solid tumor via magnetoelectrodynamic therapy. This study hints at a novel magneto-responsive nanocatalytic approach for cancer treatment. It shows prospects for future clinical translation, thanks to its excellent catalytic dynamic responsiveness, high therapeutic efficacy, and safety profile. BiOCl-based nanocatalysts formulated by Liao et al. [130] achieved wireless in situ H_2_O_2_ supply under ULS, allowing for a synergistic dual-cytotoxic effect of piezoelectricity and radioactivity. This strategy potentially overcomes the shortfall of radiocatalytic therapy associated with the insufficiency of intracellular catalytic substrates. Recently, PZE, mitochondria-targeted, ULS-triggered WS_2_ nanosheets have demonstrated promising efficacy against breast cancer [141]. PZE polarization efficiently separated the sonoexcited electrons and holes created in the WS_2_ nanosheet, promoting the generation of ROS. Incorporation of a glycolysis inhibitor to disrupt intracellular energy metabolism further augmented the therapeutic efficacy. In vivo studies also indicated localization of the NMs at tumor sites, facilitating tumor suppression without systemic effects. Herein, the remarkable combination of ULS-triggered piezodynamic cancer therapy and energy metabolism-targeted chemotherapy poses a novel, effective modality of tumor treatment. Nazir and Iqbal [136] attempted to meet the unmet clinical need for robust anti-cancer therapeutics, whereby the efficacy of injectable shear-thinning biomaterials for cancer would not be limited due to issues like stability, interaction with normal tissue, or the lack of real-time tracking. ULS stimulation of MoS_2_ nanoflowers stabilized with sheer thinning hydrogels demonstrated attractive anti-cancer activity inducing apoptosis via the caspase-3 pathway. In vivo results, too, depicted remarkable tumor reduction in mouse models within a short span of 7 days. This study provided a new piezoelectricity-based sheer thinning biomaterial with a futuristic promise regarding injectable nanotherapeutic modalities against cancer. 2D layered Bi_4_NbO_8_Br (BNB) nanosheets with remarkable PZE characteristics have been fabricated to take advantage of ULS-triggered piezodynamic therapy [142]. Under ULS stimulation, the mechanical strain of 2D stacked BNB nanosheets generates PZE polarization and band tilting, which promotes ROS generation. The BNB nanosheets lead to mitochondrial damage in cancer cells by regulating phosphorylation of the MAPK cascade. Supported by i*n vivo* observations against hepatocellular carcinoma, the study opens up new dimensions towards exogenous-mediated PZE-based tumor therapeutics.

### Biopiezoelectrics enhance drug delivery and targeted therapies

Major challenges to successful treatment of cancer are mostly related to acquired resistance to chemotherapeutic medicines and, following surgery, cancer recurrence in the presence of residual microscopic foci. Low-intensity electrical stimuli offer a physical modality to minimize resistance as well as to ensure anti-proliferative effects. It is imperative to selectively direct the therapeutics to malignant cells, preserving the healthy ones. Thus, active and passive targeting strategies become vital in cancer therapy. Table 4 represents targeted therapeutic modalities utilizing PNMs against cancer.

**Table 4:** Tumor targeting strategies utilizing PNMs

Targeting strategies	Outcomes	References
B-NPs functionalized with anti-HER2 antibody	Specific targeting towards HER2 + breast cancer	[24]
PZE B-NPs functionalized with transferrin receptor-specific antibody	Enhanced BBB penetration to treat glioblastoma multiforme	[22]
Magnetically controlled PZE nanorobot	Precise drug delivery in a time and location specific manner	[56]
Magnetoelectric NPs	Solid tumor ablation via magnetoelectrodynamic therapy	[133]
ApoE-functionalized polymeric PNMs	Passage across BBB for anti-angiogenic therapy of cerebral microvascular cells	[139]
PZE single-crystal transducer	Controlled drug release at remote regions	[55]
Pyroelectric catalysis-based nano-lymphatic	Decomposition of tumor interstitial fluid to reduce tumor interstitial pressure and enhamce tumor penetration	[132]
Au-decorated ZnO nanorods loaded with piperlongudine	Cancer-specific chemo-piezocatalytic combination therapy	[121]
ApoE-functiomalized lipid-polymer hybrids	Huge BBB permeation to treat glioblastoma multiforme	[138]
MMP2-activated PD-L1-based targeting of cancer cells with B-NPs	Immune stimulation and specific PD-L1 inhibition	[127]
Mitochondria-targeted, ULS-triggered WS_2_ nanosheets	Mitochondria-targeted, piezodynamic cancer therapy	[141]
ULS-triggered piezocatalysis to control NO	Selective generation of oxidative stress in the hypoxic tumor environment	[124]
VA-SAM-BTO	Inflammatory targeting and hypoxic targeting of cancer	[143]
Cancer therapeutic dot combined with Au-PEG-ZnO nanostructures	Wireless control over activation of electron-hole pairs from the nanocomposites	[144]

In a very interesting study regarding controlled drug delivery, a magnetically controlled PZE nanorobot has been designed to deliver doxorubicin to cancer cells [56]. The multifunctional nanorobot (nanoeel) is composed of a polypropylene head, decorated by nickel rings for magnetic actuation, and contains a PVDF copolymer tail for spontaneous piezopolarization under strain. Owing to the influence of an alternating magnetic field, the head oscillates to induce electric polarization in the PVDF tail, releasing the drug depending upon electrostatic repulsion. Such a technique prevents premature drug release from the carrier and delivers the drug precisely in a time and location-specific manner, making it a highly desirable modality aimed at clinical translation. A PZE single-crystal ULS transducer has been fabricated to achieve controlled release of drugs at remote regions of the gastrointestinal tract to treat unresectable tumors [55]. This device has also the potential to overcome the limitations of current drug delivery devices that are too large to be utilized in gastric endoscopy [145, 146]. Regarding the device, permeability could be tuned by adjusting the duty ratio of the transducer. The sonophoresis mechanism has been linked with the acoustic streaming and the thermal effect generated by the ultrasonic transducer. The PZE single-crystal ULS transducer allows for improvements in site-specific drug concentration and bioavailability [55].

Combinations of anti-cancer drugs with intrinsic EFs may be suitable options to address the inconvenience of carrying an external device or tissue damage due to the surgical insertion of electrodes. Nutlin-3a-loaded, ULS-sensitive, lipid polymer hybrid PNMs have been designed to evaluate the same against glioblastoma multiforme [138]. The nanocarrier was functionalized with ApoE peptides to facilitate BBB permeability. To trigger drug release and the local delivery of anti-cancer electrical impulses, this nanocarrier could be remotely actuated by ULS mechanical stimulation. The nanocarrier utilized chronic PZE stimulation in conjunction with chemotherapy to activate proapoptotic and anti-proliferative mechanisms in drug-resistant glioma cells, induce cell necrosis, and upregulate apoptotic and necrotic events, while preventing tumor migration and lowering cell invasiveness. This wirelessly activated electrochemotherapy approach provides new, less invasive, more focused nanomedicine strategies to remotely treat brain cancer and neurodegenerative diseases.

Similarly, Sen and co-workers [139] employed nutlin-3a-loaded polymeric PNMs to stop the angiogenic actions of human brain microvascular endothelial cells that could remotely respond to ULS stimulation. Hoang and colleagues [121] fabricated Au-decorated ZnO nanorods with enhanced ROS production abilities, loaded with the pro-oxidant agent piperlongumine to improve specificity. In another electrochemotherapy-based approach, selenium-doped PZE implants could significantly alter the permeability of cell membranes, thereby upregulating intracellular drug uptake [140]. This highlights the new use of electrochemotherapy not only to treat existing tumors but prevent the recurrence of tumors post-surgical removal. Cumulatively, both chemocatalytic and piezocatalytic techniques open many attractive horizons for cancer-specific tumor therapy.

Nowadays, nitric oxide (NO) is gaining attention to treat pancreatic ductal adenocarcinoma by regulating the dense fibrotic stroma in the tumor microenvironment to increase drug penetration [147, 148]. To address the issue of time and site-specific release and reduce systemic toxicity, Wang et al. [149] applied ULS-mediated piezocatalysis for selectively controlled release of NO along with chemotherapeutic agents. Depending on ULS-triggered piezocatalysis, BaTiO_3_ in the PZE nano-prodrug system can continuously generate stress in the hypoxic tumor environment. This allows for the release of camptothecin and oxidation of L-arginine to release NO, simultaneously delivering both to the tumor site. The success of this approach led to a big leap past the chemoresistance incurred by highly malignant pancreatic tumors, igniting a ray of hope for the future. Further, reduced tumor interstitial pressure can be accomplished by using the pyroelectric substance CdS-assisted nano-Nb_2_C MXene to improve tissue penetration [132]. Laser irradiation resulted in photothermal treatment and tumor interstitial fluid disintegration. This procedure alleviated more than half of the tumor interstitial pressure, leading to improved tumor penetration and efficient control over tumor proliferation as well as recurrence. This indicates the capability of lowering tumor interstitial pressure by splitting water. Reduced interstitial tumor pressure increases blood perfusion, enhancing the oxygen content in the tumor while aiding in the accumulation of the nanomedicine. As a surrogate marker, piezo-induced free lactate depletion in tumors indicates that lactate was bound to the positive charge of pyroelectric catalysis, hence aiding in ROS formation. During light irradiation, nanocarriers destroyed deep tumor stem cells, reducing the chance of tumor recurrence after treatment [132]. Overall, this approach gives rise to a rational for enhanced tumor penetration, which bears great importance for drug delivery.

*Staphylococcus aureus* cell membrane–coating BaTiO_3_ nanocubes on the surface of *Veillonella atypica* cells (VA-SAM-BTO) has been explored for specific targeting of orthrotropic colorectal cancer [143]. VA-SAM-BTO successfully targeted cancer through inflammatory targeting and hypoxic targeting. Under ULS excitation, the PNMs gained control over redox homeostasis, inducing immunogenic death of tumor cells. They effectively disrupted the immunosuppressive microenvironment, upregulating maturation of dendritic cells, polarization of macrophage M1 and effector T cell proportions while reducing regulatory T cell numbers. As a result, synergetic catalysis and immunotherapy could be favored. Another novel piezo-photodynamic approach has been facilitated using Au-PEG-ZnO nanostructures [144]. The nanocomposites comprising of AuNPs and ZnONPs elevates ROS generation to inhibit tumor growth. A self-powered cancer therapeutic dot, placed around the tumor tissue could wirelessly control activation of electron-hole pairs from the nanocomposites through ULS and UV radiation. The generated EF shortens electron-hole recombination, and elevates ROS induction within cancer cells. The synergistic combination of photodynamic and sonodynamic therapy, along with minimally invasive therapeutic dot explores novel perspectives in cancer therapeutics. A novel piezo-enhanced near infrared photocatalytic nanoheterojunction integrated injectable biopolymer hydrogel attempted to resolve the issue of allowing bone tissue regeneration while preventing tumor recurrence in case of osteosarcoma [150]. The nanostructures could achieve PZE-driven quick charge separation coupled with surface plasmon resonance to produce ROS. The hybrid nanotherapeutic tool could be integrated into biopolymer hydrogels, exhibiting overwhelming anti-cancer performance under the irradiation of near-IR and ULS. Hydrogels achieved very good tumor suppression ability in vivo along with good filling and retention abilities at the defective region of the bone, thus indicating repairing potential under the influence of electrical stimuli and ULS.

### b-PMs across the blood-brain barrier

**Fig. 4:**
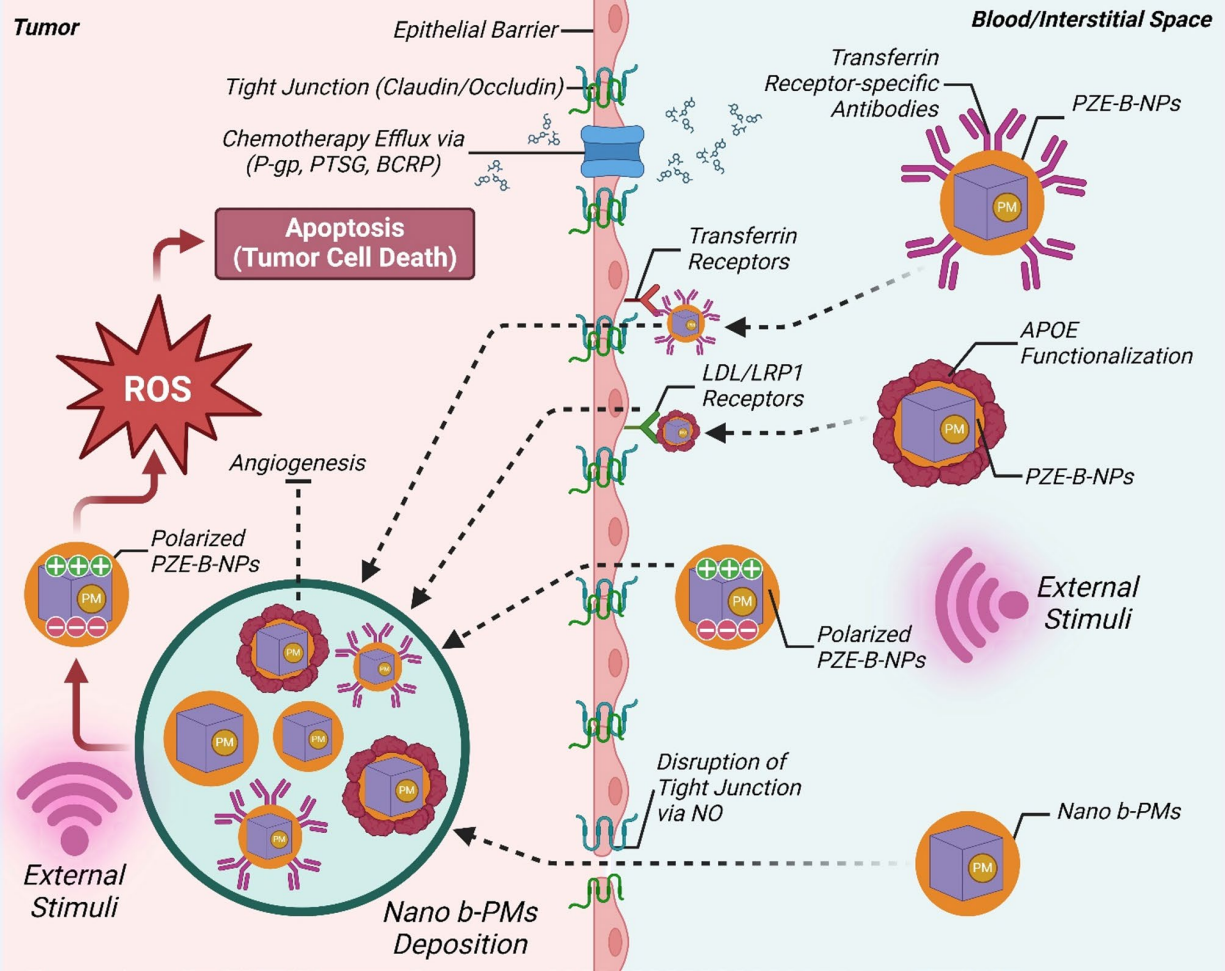
Scheme of passage of PNMs across BBB to reach CNS for cancer therapy. Endothelial cells covering the cerebral capillaries and junctional adhesion protein constitute BBB, provided with efflux transporters. NO disrupts tight junction proteins to allow ULS-guided PNMs to gain entry into CNS, polarized transferrin receptor-targeted antibody-functionalized PNMs and ApoE-functionalized PNMs gain entry through receptor-mediated transcytosis. The build-up of PNMs inside CNS leads to generation of ROS and leads to apoptosis

The transport of therapeutic agents beyond the blood-brain barrier (BBB) is a major challenge. Endothelial cells covering the cerebral capillaries along with proteins, i.e. occludin, claudin, and junctional adhesion protein, constitute BBB, a selectively permeable barrier to the brain. Efflux transporters e.g. P-glycoprotein, peptide transport system 6 (PTS6) and breast cancer- resistant protein (BCRP) hinders entry of majority of the therapeutic agents into CNS [28]. The tight junction proteins play vital role in the transfer of specific components across BBB. Charged molecules tend to gain entry faster, polar moieties might also be preferred for receptor-mediated transcytosis. Interestingly, combined effect of NO and direct current guided by ULS may allow entry of certain moieties into the CNS temporarily. The release of NO could temporarily disrupt tight junctions of the BBB, thus allowing accumulation of PNMs within brain parenchyma [151]. ULS-sensitive PZE B-NPs were designed to remotely deliver electrical signals to glioblastoma multiforme cells, sparing healthy cells [22]. To ensure passage through the BBB, the cargo was also functionalized with a transferrin receptor-specific antibody. The enhanced sensitivity toward chemotherapy, along with efficient anti-proliferative and pro-apoptotic effects established the superiority of the dual targeting strategy. In an interesting study, nutlin-3a-loaded, ULS-sensitive, lipid polymer hybrid PNMs were functionalized with ApoE peptides to facilitate BBB permeability [138]. ApoE binds with LDL receptors of the capillary endothelial cells, thus enhancing passage of the PNMs across BBB via transcytosis. Results indicate at the possibility of a less invasive and more focused PZE-based nano-system for management of glioblastoma. ApoE functionalization was also utilized successfully to attain anti-angiogenic therapy of cerebral microvascular cells with polymeric PNMs [139]. In a recent development, PNMs were camoflauged with glioblastoma cell membrane extract delivered great biocompatibility, targeting ability, and BBB crossing ability [152]. Ultrathin PZE nanosheets might potentially facilitate passive transport through BBB, aided by BBB-shuttle peptides (Trojan horses) [153]. Different strategies implied for passage of PNMs across BBB have been represented in Fig. 4.

### Biopiezoelectrics with tunable catalytic properties

Piezo-enzymes utilize PZE materials to transform mechanical energy into electrical energy, thereby initiating enzymatic activities. Piezo-enzymes can be engaged to specifically kill tumors by combining the benefits of piezoelectricity and enzymatic catalysis. In the context of PNMs, piezoenzymes are individual PMs capable of acting as nanozymes themselves [154]. Experimentally, PZE potentials were induced on MnTiO_3_ nanosheets only when ULS waves triggered the nanozymes to commence the enzymatic reaction with surrounding glutathione, thus avoiding unwanted effects to normal tissues [135]. Furthermore, the peroxidase-mimetic and catalase-mimetic functions of MnTiO_3_ nanosheets changed with pH. By utilizing ULS and tumor microenvironment in combination with the controllability of multi-mimetic enzyme activity, the scientists put forward a novel idea of programmed tumor destruction. Attempts to precisely control the catalysis of enzymes via tuning their temperature-dependent kinetics are also feasible. Experimentally, cold-activated artificial enzymes have been produced with Bi_2_Fe_4_O_9_ nanosheets [129], which exhibit glutathione oxidase-like activity at low temperatures. Bi_2_Fe_4_O_9_ nanosheets lead to tumor cell death under cold conditions by inducing apoptosis and ferroptosis, while minimizing off-target damage [129]. Furthermore, an interventional device has been developed to remotely control the enzymatic activity of the nanosheets using a smartphone. This strategy bears potential for cancer vaccines that can provide precise control over piezocatalytic nanomedicines. Under ULS stimulation, monolayer MoS_2_ nanoenzymes demonstrate PZE polarization, generating ROS that inactivate malignancies [155]. However, due to stacking of MoS_2_, quenching of piezo-generated charges, and monotherapy, the therapeutic effectiveness is not up to the mark. Chitosan-exfoliated monolayer MoS_2_ composited with atomic-thin MXene to self-assemble into a multi-modal nanoplatform has been formulated to preserve the anti-tumor activity [137]. Apart from inheriting piezoelectricity from monolayer MoS_2_, it also exhibits excellent stability along with negligible toxicity to normal tissues. MXene, an intrinsic metallic compound, intermixes with MoS_2_ to form an interfacial Schottky heterojunction which leads to peroxidase mimetic activity towards H_2_O_2_ in the tumor microenvironment. This work bears the potential to shed new light on optimizing PZE performance in biological applications, simply by allowing for new combinatorial chemistry. Sulfur-doped graphdyine nanozymes conferred synergistic ferroptosis-apoptosis effect on triple-negative breast cancer cells [124]. It could convert H_2_O_2_ to harmful hydroxyl radicals and induce apoptosis. More critically, the ULS-regulated nanozyme triggered ferroptosis by endorsing an unbalanced redox response caused by glutathione depletion and glutathione peroxidase 4 inactivation. Importantly, it could effectively induce ferroptosis-apoptosis to exert tumor therapy in mouse models.

### Role of O_2_ in biopiezocatalysis

**Fig. 5:**
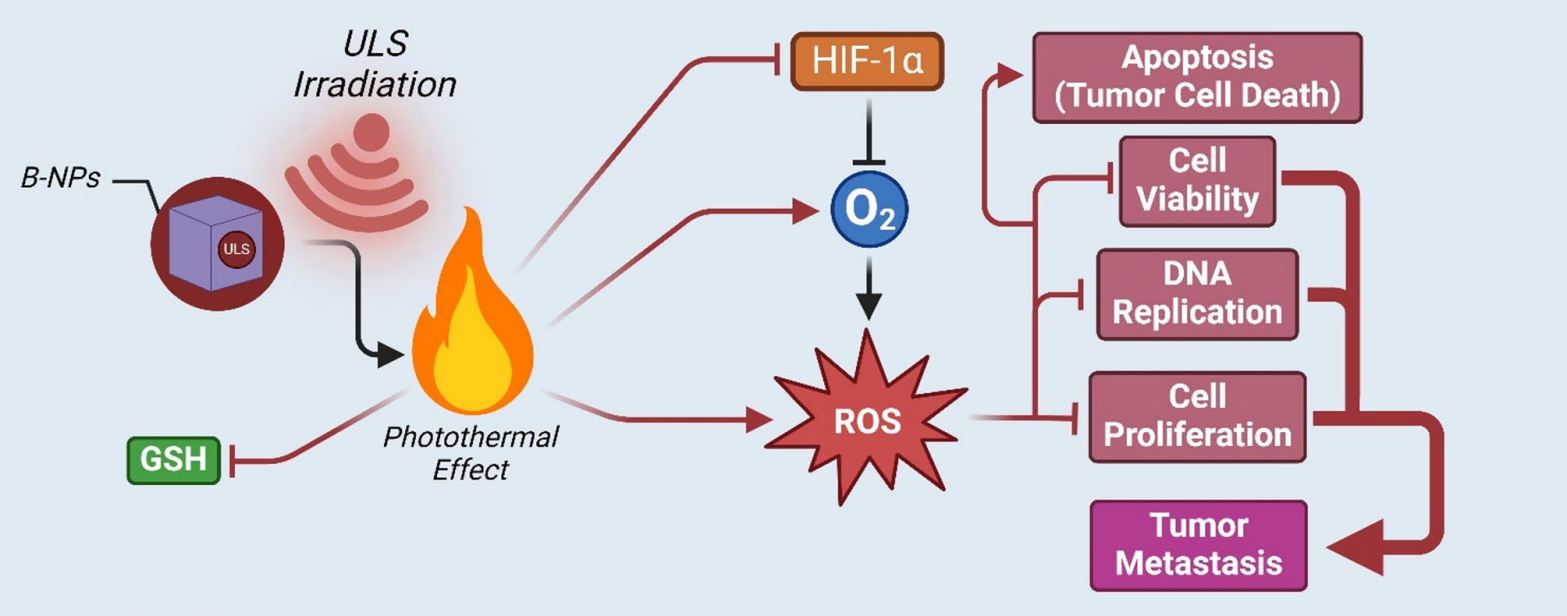
O_2_-dependent catalytic activities by anti-cancer b-PMs. Overexpression of HIF-1α in tumor microenvironment leads to hypoxia, which suppresses ROS generation in the tumor cells. This increases cell viability, DNA replication and rate of proliferation while decreasing apoptosis leading to possible aggravation and metastases. B-NPs present at the tumor location, when irradiated with ULS, generates a photothermal effect which depletes antioxidant enzyme GSH, depletes HIF-1α, leading to increased oxygen availability, and causes ROS burst thus inhibiting metastases and accelerating apoptosis

PZE dynamic therapy often results in inadequate efficiency since the slow oxygen diffusion rate fails to keep up with the high oxygen consumption rate, despite plentiful research into the design of devices that aid in the tumor-specific distribution of oxygen. Ding and colleagues [125] attempted to resolve this issue by fabricating oxygen-vacancy-modified NPs for an oxygen-independent dynamic treatment approach. Under hypoxic conditions, oxygen-independent PZE-mediated photothermal treatment causes significant damage to tumor cells to inhibit tumor growth. As already discussed, hypoxia is a characteristic trait of tumor microenvironment and is associated with the overexpression of HIF-1α, a protein commonly correlated with tumor metastasis [156]. Since hypoxia impedes the generation of ROS, it is important to increase the oxygen concentration at the tumor site to improve the success of anti-tumor therapies. Wang and peers [126] fabricated DSPE-PEG2000 coated B-NPs tailored for the hypoxic state of tumors, which can kill cancer cells by virtue of PZE_e_. During ULS irradiation, the ultrafine NPs develop an imbalanced surface charge, starting a redox reaction and producing both oxygen and ROS. As a result of the in situ oxygen synthesis, hypoxia in the tumor microenvironment may be significantly eased, reducing the expression of HIF-1α, inhibiting tumor growth, angiogenesis, and metastasis. Analogously, thermally treated natural sphalerite PZE nanosheets successfully led to a ROS burst, culminating in the apoptosis of breast cancer cells [32]. The piezo-photocatalytic effect led to efficient catalysis, resulting in high production of superoxide radicals (˙O_2_^−^) and hydroxyl radicals (˙OH), and depletion of GSH. Consequently, intracellular ROS burst, followed by apoptosis of cancer cells were achieved both in vitro and in vivo. Fig. 5 schematically depicts oxygen-dependant catalytic roles of biopiezoelectric NPs.

Under ULS irradiation, the incredible piezoelectricity of KNN NPs generates a massive built-in EF that changes constantly. This permits holes and electrons to be continually separated, resulting in redox processes stimulated by piezoelectricity. Furthermore, KNN is typically riddled with point defects such as oxygen vacancies, making chemo-catalytic activity a viable alternative for treating malignancies [157]. On this basis, Wang and co-workers [158] proposed KNN (lead-free) PNMs to possess both chemical catalytic effects driven by oxygen vacancies and ULS-triggered PZE catalytic effects. The KNN nanocrystals exert obvious toxic effects on tumor cell growth under the synergistic chemical-piezoelectric catalytic effect, and can effectively induce apoptosis, and inhibit the proliferation of osteosarcoma cells. The anti-tumor effect has been supported through in vivo models devoid of any observable effect on normal cells and organs. This study integrates synergistic catalytic effects for therapeutic applications in tumor treatment, opening up a new channel for the personalized design of high-tech nanocatalysts. In a recent study, heterostructured nanorods emphasized on elevating ROS production, thereby enhancing piezocatalysis [159]. The oxygen vacancies on the surfaces give rise to electron-rich sites, which substantially increase the piezocatalytic abilities. Both in vitro and in vivo findings on hepatocellular carcinoma establish the anti-tumor activity of the PNMs, thereby pleading for this as a promising tool against cancers.

### PNMs and immunotherapy

Immune cells are assigned to tumors to suppress tumor growth. However, hypoxic tumor microenvironment leads to less availability of immune cells in the vicinity of tumor tissues [160]. Therapeutic transfer of T cells to tumor location is a therapeutic strategy coined as adaptive immunotherapy [161]. PNMs have been reported to relieve hypoxia and restore oxygen supply in tumor microenvironment when exposed to external stimulation [162]. Nanopiezoelectric immunotherapy involves activation of the immune system via regulation of electrical signals [163]. Kong and co-workers [59] used ULS to non-invasively boost macrophage proinflammatory responses via local electrical impulses mediated by PMs. This technique is based on the microvibrational deformation of PZE β-PVDF films by ULS irradiation, resulting in the release of local charges following spontaneous crystal phase polarization. These local electrical signals could be detected by voltage-sensitive receptors on macrophages, consequently leading to the selective generation and/or secretion of proinflammatory chemoattractant moities. Interestingly, the electric potential, not ROS, is the active driver of the process, confirmed by omics studies thus establishing the importance of electrical stimuli in cancer therapeutics. Tumor immunotherapy based on immune checkpoint blockage still suffers from a low host response rate, while the non-specific distribution of immune checkpoint inhibitors severely limits the therapeutic efficacy. Artificial exosomes expressing surface αPD-L1 derived from genetically engineered cells with an MMP2-activating lock masking on αPD-L1 were used to encapsulate B-NPs and amplify immune response by selectively blocking the PD-1/PD-L1 cascade at tumor sites [127]. With ULS irradiation, PNMs can simultaneously produce ROS and oxygen, based on BaTiO_3_-mediated piezocatalysis and water splitting, respectively. This approach provides a robust, safe way of immune stimulation, significantly promoting the intratumoral infiltration of cytotoxic T lymphocytes and improving PD-L1 blockade therapy to tumors. In a mouse model, the nanoplatform effectively connects MMP2-activated genetic editing cell membrane with ULS-responsive B-NPs for both immune stimulation and specific PD-L1 inhibition, thus allowing for a safe and robust strategy to uplift immune response against tumors. Wang and colleagues [162] formulated BiVO_4_ nanorod-supported platinum nanodots which is a piezoelectric-photothermal sensor that can catalyze dissociation of hydrogen peroxide and release oxygen in the tumor microenvironment under ULS stimulation. This reverses the immunosuppressive microenvironment with increasing oxidative stress. In another experiment. Hao and co-workers [164] formulated BaTiO_3_/(Cp^*^RhCl_2_)_2_ nanoparticles with doxorubicin. After delivery, upon ULS stimulation, intratumor infiltration of cytotoxic T lymphocytes is enhanced leading to cytotoxicity.

### Biopiezoelectrics improve classical techniques for anti-cancer therapy

Dynamic treatments use ROS to kill target cells, showing promise for tumor treatment since they transcend the limitations of chemotherapy approaches. PMs convert mechanical energy into electrical energy to drive chemical reactions and generate ROS, leading to the development of ULS-driven tumor therapy techniques. PNMs are intended to provide a more efficient and safe option for cancer therapy with improved patient compliance. Electric signals generated by smart PZE scaffolds treat affected tissues through diverse approaches.

The development and clinical evaluation of ablation needles is presently in its early days, with PMs serving as transducers in most approaches [8, 165]. Despite being therapeutically acceptable, thermal ablation has limitations when the tumor is close to important blood vessels because of the possibility of severe thermal collateral damage. An effective non-thermal tumor ablation method in this instance is electrolytic ablation, which selectively kills cancerous cells by causing pH changes in the immediate environment [166, 167].

Tumor tissues frequently produce pathological blood vessels, which cause local hypoxia and reduce blood transport efficiency, impeding drug transport and therapeutic effects within tumors. Li and colleagues [128] developed tetragonal-polarized BaTiO_3_ PNMs with high electromechanical conversion efficiency toward electrical stimulation-induced normalization of tumor blood vessels under ULS. Furthermore, the combination of polarized NPs with doxorubicin demonstrated remarkable anti-cancer benefits. Wireless electrical stimulation blocks migration and differentiation of endothelial cells. The blockade of angiogenesis-related eNOS/NO cascade could be attributed to the severe reduction of intracellular Ca^2+^ gradient. In vivo studies indicated that electrical stimulation normalizes tumor vasculature by revising vascular structure, increasing blood perfusion, minimizing vascular leakage, and restoring local oxygenation. This study proposes a wireless electrical stimulation technique based on the mechano-electrical activity of PNMs, with the goal of achieving safe and effective clinical adjuvant treatment of malignant tumors, chiefly by breaking the bioelectric homeostasis within cancer cells. In a novel development, electroactive microenvironments have been utilized by PVDF-based nanofilm to mitigate rhabdosarcoma [21]. The findings hint at the possibility of utilization of electroactive microenvironments to develop novel approaches to deal with rhabdomyosarcoma, that may be expanded to other cancer types. Though it pioneers a new strategy for tumor eradication, more detailed mechanistic insights are required to convincingly decide on its developmental potential.

Atomically thin PZE nanosheets reduce the migration distance of carriers, elevate the specific surface area, and improve charge transfer efficiency, exploring natural advantages towards ROS production. The non-centrosymmetric polar crystal structure allows the nanosheets to segregate electron-hole pairs. Under ULS-mechanical stress, nanosheets with PZE feature present with ROS bursts to lead to Caspase-1-driven pyroptosis, displaying acceptable in vivo anti-tumor outcomes [168]. In a very interesting development, PVDF/AgNPs nanocomposites enhance the PZE signal along with improved biological activity [169]. The PNMs exhibited cytotoxic potential towards triple negative breast cancer cells while normal cells remained unaffected. Thus, the nanocomposites might provide a breakthrough in the safe management of triple negative cancer, an otherwise difficult-to-treat subtype of breast cancer. MoS_2_-based nanoflowers utilized piezo-sonosensitization by introducing sulfur vacancies [170]. Improved PZEe could be attained under mechanical stress for cancer therapy. ROS production could be improved to enhance catalytic performance. Adding the in vivo anti-cancer activity with biocompatibility, this vacancy-engineering strategy allows for a novel technique to elevate sonodynamic performance of PNMs.

### Combination therapy

PNMs exhibit promise regarding combination therapy with chemotherapeutic agents against cancer. The potential of PNMs facilitating different therapeutic attributes has been discussed in earlier sections. Barium-based NPs combined with anti-HER2 targeting have shown synergistic anti-tumor activity against HER2 positive breast cancers [24]. Liao et al. [130] formulated BiOCl-based nanocatalysts to obtain in situ wireless H_2_O_2_ supply under ULS, and applied the same in combination with radiotherapy. The combination shows enhanced cytotoxicity against cancer cells and somewhat overcomes the concerns associated with radiocatalytic therapy by supplying intracellular catalytic substances. ULS-sensitive PNMs have loaded with nutlin-3a and functionalized with ApoE peptides attempting to treat glioblastoma multiforme [138]. The formulation exhibited stimulus-dependent drug release, enhanced BBB penetration and induced cell necrosis, apoptosis, and activated antiproliferative pathways in drug-resistant glioma cells [138]. This electrochemotherapy based approach has also been adopted in another study whereby a prooxidant piperlongumine has been combined with gold-functionalized ZnO nanorods [121]. Attempting to treat pancreatic ductal carcinoma, B-NPs based nano-prodrug system loaded with camptothecin have been developed [149]. When ULS is used to trigger the piezocatalysis, the hypoxic tumor microenvironment undergoes stress, camptothecin is released in the tumor site and oxidation of L-arginine facilitates the release of NO [149]. The combined effect led to optimistic promise in treatment of malignancies related to pancreas. In a recent study, doxorubicin-loaded hexagonal boron nitride nanosheets have been delivered to human prostate cancer cells; the results showed ULS-stimulated formulation imparts significant decline in proliferation of the cancer cells while inducing both early and late apoptosis, and achieving this with much less concentration of doxorubicin [171]. Endoplasmic reticulum targeted PNMs have displayed novel immune checkpoint blockade in case of triple negative breast cancer [172]. These findings clearly indicate that PNMs hold promising ability to potentiate other conventional modes of therapies to demonstrate synergistic enhancement in the therapeutic outcome.

## Current challenges and prospects

Despite their immense promise, preparing good quality b-PNMs with well-defined morphologies remains a considerable challenge. Most biopiezoelectric devices often place high demands regarding the flexibility of the material. The administration of PNMs in vivo aims to treat disease condition with minimal effect on healthy tissues, therefore the toxicity evaluation and assessment of adequate administration rates is a vital factor that requires careful examination. Site-specific targeting might provide a potential solution to it (Table 4), however, precise control over the overproduction of ROS is a critical challenge [173]. The exact pathway/s of cell responses toward PZE stimulation is/are still not uncovered fully [174, 175]. There are multiple factors (size of NPs, type of cells under investigation, mode of functionalization, etc.) that might affect the internalization. Furthermore, the accurate measurement of PZE_e_ of NMs is an arduous process.

Though piezoresponse force microscopy is widely accepted method to detect and quantify PZE behavior of NMs, PZE_e_ measured through this method usually gets influenced by electrostatic attraction between the sample surface along with the resonance frequency related to the cantilever’s tip [176]. Consequently, this might adulterate and provide incorrect results. Hence, novel techniques with more accurate dynamic measurements are in demand. The majority of b-PMs research has been on investigating and improving material physical qualities e.g., shape, transparency, flexibility, piezoelectricity, and mechanical strength. The chemical characteristics of such, as well as the specific interactions between biological systems and piezoelectricity, have received comparatively less attention. Nowadays, ULS is the most common mechanical stimulus used to generate piezoelectricity [177]. Yet, a high dose of ULS can cause tissue damage due to ULS cavitation effects [178]. As a result, alternative biocompatible techniques for inducing material piezoelectricity may have to be adopted in some cases. Interestingly, increasing the piezoelectricity of existing b-PMs can reduce the level of applied ULS power within biologically safe levels [110]. Moreover, improving the efficiency of energy conversion from external stimuli to electrical signals is still a challenge to be addressed. Another aspect that currently restricts the biomedical application of PNMs is the difficulty in separating and excluding cellular responses to non-specific phenomena (mechanical/thermal) that could display an overlapping impact with electrical stimuli. This could lead to confounding variables impacting the interpretation of efficacy studies, directly impacting assay confidence towards clinical translation.

Sufficient flexibility and durability are two factors that frequently limit the utilization of ceramic-based b-PNMs. To overcome the same, B-NPs are frequently composited with matrices of various soft polymers and their derivatives [179]. Another promising b-PM, ZnO, suffers from several concerns, primarily the screening effect, which hinders its PZE performance [180]. Contextually, fabricating different morphologies is considered an excellent approach for improving the performance of ZnO-based nanogenerators [71].

The biochemical pathways involved in the organelle-specific functionalization of b-PMs call for an in-depth investigation. The current spectrum of available materials for b-PMs is still restricted, showcasing a true need for further research of new PZE and ferroelectric materials/composites, as well as their surface modifications. Interestingly, lead-free ferroelectric ceramics, polymers, and composites could be very helpful in this area [181]. Of particular interest is the potential for the involvement of high-throughput and machine-learning techniques in future discovery [15]. Clinical application in tumor therapy is the primary goal of b-PMs. Though b-PMs have exhibited early promise in the field, their long-term toxicity and safety, targeting ability, and biodegradability require further investigation [33]. The development of b-PMs with high PZE effectiveness is very desirable to reduce the required dosage. Gallium nitride, with its unique PZE, pyroelectric, and piezoresistive properties, has the potential to become an efficient tool to manipulate cancer cells [182]. Furthermore, optimizing synthesis processes and developing new surface engineering technologies are critical for advancing towards clinical translation of b-PMs. A potential solution and new avenue for b-PMs are hybrid nanogenerators, which harvest energy from multiple sources concurrently, significantly improving output performance relative to classical monogenerator PZEs [71, 183].

While b-PNMs exhibit potential in cancer therapeutics, there still exist concerns to their rampant utilization. These include successfully delivering the platform to the target region, ensuring the stability and long-term performance of the nanoplatforms regarding its safety and biocompatibility, and immunogenicity. Furthermore, regulating the responsiveness to stimuli and maximizing interaction with the tumors are critical for attaining the intended therapeutic result. Complexities of target tumor tissues, especially the tumor microenvironment and protein corona formation may hinder accurate delivery of the nanoplatforms [8, 152]. Further, factors like extracellular matrix and vascular barriers might potentially restrict delivery of nanoparticles to tumor regions. Revisiting formulation strategies, without robbing off the major advantage of non-invasiveness of b-PMs might aid in improving the target efficiency for site-specific delivery. Moreover, It is imperative to ensure the PNMs stay stable and operational within the body over time. Thus, the PMs must be safe and biocompatible without causing any major adverse reactions. Again, some b-PNMs may pose with the risk of being recognized as foreign in the body, and consequently may trigger immunological response [6], which may include inflammation, generation of antibodies, and activation of immune cells. The material of construction and surface properties critically control the immunogenicity. The size and structure of the nanoplatforms can influence how it is recognized and handled by the immune system, While immunogenicity is a concern, it can also potentially be employed for therapeutic purposes, such as in cancer immunotherapy, whereby PMs might improve macrophage polarization and suppress the growth of tumor cells.

Malignant tumors largely suffer from poor prognosis, along with short average survival post-diagnosis [184]. Major variables limiting the efficacy of treatment, which often involve surgery followed by chemotherapy and/or radiotherapy, include drug resistance developed during chemotherapy and disease recurrence initiated by the remnants of microscopic tumor foci [185]. Researchers are working on PNMs that target and electrically excite tumor cells to improve the treatment of different malignancies. PMs have attracted overwhelming attention in the field of oncology because of their electromechanical coupling ability. The electrical signals generated by b-PMs are used for redox reactions and drug delivery, among others. Compared to normal cells, cancer cells exhibit an exaggerated oxidative stress response when exposed to enhanced ROS levels. Thus, cancer cells are more sensitive to agents that increase oxidative stress [186]. Moreover, PNMs are exhibiting promising outcomes in animal models also (Table 5). Thus, there exists a clear ray of hope regarding clinical translation of biopiezoelectrics to cancer therapeutics.

**Table 5:** In vivo anti-cancer effect of b-PMs

Formulation types	Disease model	Observations	References
Magnetoelectrodynamic NPs	Tumor xenograft model (triple-negative breast cancer in mouse)	Significant tumor regression	[133]
Nanosheets	Breast cancer mouse model	Tumor suppression by ROS-mediated apoptosis	[32]
BaTiO_3_ PNMs	Mouse melanoma model	Revision of tumor vasculature	[128]
MoS_2_ nanoflowers	Mouse model of skin cancer	Tumor reduction within a short timespan	[136]
KNN PNMs	Mouse osteosarcoma model	Tumor eradication by chemo-piezo catalysis	[158]
Immunotherapeutic B-NPs	Mouse skin cancer model	Tumor-specific immunotherapy	[127]
WS_2_ nanosheets	Tumor xenograft model in mice	localization of the NMs at tumor sites, facilitating tumor suppression	[141]
Sulfur-doped graphdyine nanozymes	Mouse model of triple negative breast cancer	Induction of apoptosis-ferroptosis for tumor therapy	[124]
MoS_2_-based nanoflowers	Mouse xenograft model of triple negative breast cancer	Very good tumor inhibition	[170]
Nanorods	Hepatocellular carcinoma	Significant tumor inhibitory capacity	[159]
Nanosheets	Breast cancer and liver cancer models	Antineoplastic effect	[168]
Piezo-enhanced near infrared photocatalytic nanoheterojunction integrated injectable biopolymer hydrogel	Mouse model of tibia osteosarcoma	Tumor suppression and bone repair	[150]
BNB nanosheets	Mouse model of hepatocellular carcinoma	Inhibition of cancer growth	[142]
BaTiO_3_/(Cp^*^RhCl_2_)_2_ nanoparticles	Mouse model of solid tumor using cervical cancer cells	Piezocatalytic chemo-immunotherapy-mediated disappearance of solid tumors	[164]
Endoplasmic reticulum targeted PNMs	Mouse model of triple negative breast cancer	Immune checkpoint blockade leading to cancer therapy	[172]

## Clinical outlook

Electrical stimulation holds a significant promise for biomedical applications. The use of PZE materials in nanomedicine for anti-tumor therapy can expand the scope of electrical stimulation in therapeutics. PZE materials transform energy from external stimuli into electrical impulses, enabling local wireless control and minimally invasive treatment options. The development and utilization of PZE materials as smart nanomedicine is still in its infancy. PZE devices offer an array of clinical applications as diagnostic tools [6]. Moreover, electrical stimulation is well-recognized to promote wound healing [187].

However, there still exist a few challenges in the clinical translation of PNMs regarding cancer chemotherapy [8]. To achieve accurate measurement of PZE properties of NMs and satisfactory energy conversion efficiency from external stimulus to electrical signal are among the major concerns. Although pressure electric microscopy with its high resolution and non-destructive imaging holds promise regarding local measurement of PZE properties, but lack of robustness and low accuracy for NMs somewhat subsides its appeal. Energy conversion involves complex phenomena that makes it difficult to predict. Additionally, the response of the test subject to PZE stimulation is not very clear since biochemical pathways affected by the PZE effect are not well-defined. Also, long-term biological safety is to be minutely assessed to ensure an acceptable risk-benefit ratio for human subjects prior to rampant clinical utilization. Biological safety parameters must be extensively studied before progressing to clinical stages. Majority of the earlier research has focused on improving dynamic therapeutic effects, which rely on electron-hole interactions and/or positive and negative polarized charges to produce ROS [17, 188]. The complexity of the tumor microenvironment, which has previously been somewhat overlooked, seems to be the root cause of the apparent failure of PNMs under clinical setup so far. However, it is imperative that PNMs, with their remarkable therapeutic potential against different cancer types in preclinical studies, do represent a notable promise regarding possibilities of future clinical translation,

## Perspectives

Sonodynamic therapy is commonly used for the minimally invasive treatment of deep tumors, whereby apoptosis through the generated ROS further aids anti-tumor therapy with PNMs. ULS activates PZE properties of NMs deep within tumor tissues by virtue of both cavitation and PZE mechanisms. This, in turn, leads to the generation of cytotoxic ROS, causing irreversible damage to cancer cells. Moreover, ULS can modulate the redox capacity of semiconductor materials by tilting the energy bands. Inspired by initial research results, the construction of smart closed-loop anti-tumor systems based on PMs in cancer therapeutics has become a futuristic development trend. Interestingly, force, acoustic, magnetic, thermal, and electrical properties can be successfully integrated into PMs, thus offering a solid material foundation for intelligent therapeutic systems. Even so, the medical applications of the developed b-PMs have mostly remained at the proof-of-concept level. Further exploration regarding research and practice is required to replicate their exceptional performance in more complicated biological systems. The first question is whether present PMs are safe for biomedical applications, and the second is whether their qualities are realistically applicable. Furthermore, the relationships between drugs, external stimuli, and the biological system must be clarified, as well as their pharmacological applicability within the preclinical and clinical phases. Also, the concerns such as delivery, tissue targeting, stability, biosafety, and immunogenicity of b-PNMs must be completely addressed prior to rampant clinical utilization.

The study of PMs as anti-cancer therapeutics is still in its early stages, and the relationship between b-PMs and biological systems is still uncertain. Most findings to date have focused on the enhancement of dynamic therapeutic effects, which rely on interactions involving electrons and holes or positive and negative polarized charges to generate ROS. The fundamental reason why most nanomedicines have failed in clinical trials seems to be the complexity of the tumor microenvironment, which has generally not been considered. The unique features of PZE, pyroelectric, and ferroelectric materials make wireless, less invasive therapies possible. Yet, the sensitivity, strength, and stability of the materials' response after entering the body are critical challenges in realistic applications. Most pyroelectric materials, for example, require a rather high rate of temperature change to transform thermal energy into chemical energy [189]. However, it is difficult to meet these temperature conditions by living organisms. Therefore, it is necessary to either construct biopiezo/pyro/ferroelectric materials with improved, tunable electromechanical coupling properties and/or energy conversion efficiency. The advancement of PZE, pyroelectric, and ferroelectric materials in nanomedicine for anti-tumor therapy bears the potential to increase the application range of electrical stimulation therapy. PMs possess the ability to transfer energy in the form of force, light, electricity, magnetism, and heat into electrical impulses, allowing for local wireless control and minimally invasive treatment techniques. This overcomes the constraints of existing electrical stimulation, which necessitates the use of connectors and batteries.

Tumor types that express large levels of specific receptors or proteins, or those that are very sensitive to mechanical stimulation, may benefit the most from b-PMs. Some tumors tend to be more sensitive to mechanical effects of external forces, e.g. ULS, they may potentially be more susceptible to PNMs. Further, b-PNMs can be tailored to target specific types of solid tumors by conjugating ligands and/or antibodies that recognize distinct cell surface antigens. Brain tumors may be suitable for PNMs-mediated therapeutic strategies owing to the ability to be affected by external forces, and the potential for localized treatment approaches [190]. The ability of b-PNMs to reach beyond BBB further approves the possibility. The b-PNMs interact with tumor cells, altering their behaviors regarding growth, proliferation, and apoptosis. Breast cancer cells often express sensitivity to mechanical stimuli, thus may undergo targeting by PNMs formulated to halt cancer progression. Emerging evidence suggests that PNMs activated by ULS, can effectively restrict the proliferation of breast cancer cells and eventually minimize volume of tumors [24, 191]. Osteosarcoma also exhibits good responses to PNMs regulated by ULS, mainly due to the increased ROS generation, and targeted delivery [31, 192]. Apart from specific tumor types, ULS-regulated b-PNMs can also be engineered to target hypoxic tumor microenvironment [193] to generate ROS. In addition to ROS production, the capability to improve drug delivery pattern, regulate the tumor microenvironment, and trigger cell death.

In many cases, novel therapeutic modalities based on PZE and pyroelectric materials utilize the synergistic effect of multiple therapeutic modalities, e.g., piezodynamic + ULS dynamic therapy, piezodynamic + ULS + photodynamic therapy, piezodynamic + chemodynamic therapy, pyroelectric dynamic + photothermal therapy, and piezo/pyrodynamic + chemotherapy. These therapeutic modalities are complementary and mutually reinforcing, aiming to achieve a more efficient, multifunctional anti-tumor effect. The urgent questions that for now restrict widespread exploitation of novel PNMs for biomedical applications are related to the chances of long-term adverse effects, their metabolic fate within the biological system, and the careful evaluation of the acceptability of their risk-benefit ratio. As discussed, the piezotherapeutic processes rely on adsorbed oxygen and water to generate ROS. In strong opposition, the hypoxic and necrotic zones within tumors may be more than half of the tumor volume, likely hindering proper oxygen supply. Despite research into the creation of devices that can produce or distribute oxygen at the tumor site, PZE dynamic therapy typically results in suboptimal efficiency due to low oxygen availability. In comparison to classical techniques, b-PMs mostly exhibit superior results, for overcoming the constraints of existing electrical stimulation i.e. the use of connectors and batteries. Utilization and integration of newer techniques like tumor treating fields, biotherapeutic devices, and mobile health hold immense futuristic potential regarding biopiezoelectric-mediated tumor treatment [194]. Moreover, b-PMs also hold promise in the prospering arena of personalized biomedicines.

## Conclusion

Since their discovery, PMs have been used in a wide range of applications. PMs allow for exciting and strong couplings between mechanical strain, light, electricity, magnetism, heat, and bring forth novel research frontiers for anti-tumor therapeutic explorations. The current PZE dynamic therapy paradigm is still in its preliminary stages, with some proven examples showing the feasibility of the therapeutic modality. The efficacy of the developed systems is encouraging, facilitating further research and implementation of dynamic therapy in cancer. In this review, we described the theoretical underpinnings of b-PMs as nanomedicines and nanocarriers, provided a comprehensive summary of techniques to enhance their PZE properties, and reviewed their current biomedical applications for cancer. Nowadays, prime importance has been placed on fabricating flexible, reliable b-PNMs for tumor treatment. b-PMs must provide a high output voltage and current as well as a long and continuous operating life, both of which may be attained with approaches such as morphological tuning, doping, and creating composites. Futuristic trends seem to focus on PZE catalysis for the treatment of tumors in situ or patient-derived tumor xenografts, which has important implications for potential future clinical translation. Overall, biopiezoelectric-mediated systems appear to hold immense promise for safe and effective treatment of malignant tumors.

## Data Availability

No datasets were generated or analysed during the current study.
